# Postmastectomy Breast Reconstruction With the Totally Autologous Latissimus Dorsi Flap in the Thin, Small-Breasted Woman: Give It More Thought!

**Published:** 2018-02-26

**Authors:** Fawz Kazzazi, Rosanna C. Ching, Charles M. Malata

**Affiliations:** ^a^Clinical School of Medicine, University of Cambridge, Cambridge, United Kingdom; ^b^Department of Plastic and Reconstructive Surgery, Cambridge University Hospitals NHS Foundation Trust, Cambridge, United Kingdom; ^c^Cambridge Breast Unit, Addenbrooke's Hospital, Cambridge University Hospitals NHS Foundation Trust, Cambridge; ^d^Anglia Ruskin University School of Medicine, Cambridge and Chelmsford, United Kingdom

**Keywords:** breast reconstruction, latissimus dorsi, autologous reconstruction, mastectomy, breast cancer

## Abstract

**Introduction:** Thin women have fewer autologous tissue breast reconstructive options than their higher body mass index counterparts—due to a lack of adequate donor sites. They are therefore usually offered expander/implant techniques. The total autologous latissimus dorsi flap is generally used in “well-padded” individuals, as they have enough fat on their back on which a completely autologous reconstruction could be based. When implant-based reconstruction is contraindicated (for instance due to planned adjuvant radiotherapy) or unacceptable to the patient, the total autologous latissimus dorsi flap can provide adequate tissue volume by utilizing the additional back fat deposits even in the thin, small-breasted patient. This option is often overlooked by many surgeons. Our case series assesses indications and patient and surgeon satisfaction with the cosmetic outcome of this technique. **Methods:** The oncological and clinical details of 6 patients with breast cancer who underwent total autologous latissimus dorsi myocutaneous flap immediate breast reconstruction by a single surgeon over an 8-year period were reviewed. An objective assessment of satisfaction with the cosmetic result was made by whether any additional surgical interventions (ipsilateral fat grafting/implant augmentation or contralateral liposuction/ reduction) were needed or not. A subjective assessment of breast symmetry by the surgeon using photographic records was also undertaken. The aesthetic outcomes were also objectively quantified using the BCCT.core software, initially developed for assessing the results of breast conservation surgery. **Results:** All 6 patients had small breasts and a low or normal body mass index. The mastectomies were performed for invasive carcinoma (n = 3) and extensive high-grade ductal carcinoma in situ (n = 3). Four had axillary surgery (2 sentinel lymph node biopsies and 2 axillary clearances), and 3 received adjuvant radiotherapy. All were happy with their reconstructive outcomes, and none suffered major postoperative complications or disease recurrence. None requested or needed any subsequent ipsilateral adjustment or contralateral symmetrizing procedures. Subjectively, the reconstructions provided acceptable or excellent cosmetic results. The cosmetic results were categorized as excellent or good on the BCCT.core scoring system. **Conclusion:** This underutilized method of totally autologous breast reconstruction in thinner patients with lower body mass indexes yielded good, well-accepted cosmetic results without recourse to adjustment procedures, contralateral balancing surgery, or complex microvascular surgery. We recommend that the total autologous latissimus dorsi flap should be given more consideration when planning immediate breast reconstruction in this challenging group of thin, small-breasted patients.

Immediate breast reconstruction following mastectomy for cancer can utilize implants, autologous tissue, or a combination of both.^1^ Recent innovations include the use of simultaneous fat grafting, combination free flaps, and acellular dermal matrices (ADMs). The key considerations in technique selection are the breast size versus the potential donor sites, the patient preference, and whether adjuvant radiotherapy (RT) is likely or not. It is often thought that the thin, small-breasted woman has more limited reconstructive options than her larger-breasted, higher body mass index (BMI) counterpart with greater fat deposits.^2^^,^^3^ This is due to a lack of donor sites on which a completely autologous reconstruction could be based. They are therefore often offered implant-based reconstructions. A common—but increasingly less used^4^—option for these women is an autologous reconstruction (latissimus dorsi [LD] myocutaneous flap) combined with an implant/expander and to symmetrize the contralateral breast either concomitantly or subsequently, if required. For thinner women with lower BMIs who refuse prosthetic reconstruction or contralateral balancing surgery, their management can be more challenging. Within the United Kingdom, the majority of females have relatively high BMIs (>25), with the average female BMI at 26.9.^5^ The postoperative need for RT also has a significant impact on the choice of reconstruction, the necessity of which may preclude implant-only reconstruction as an option anyway, due to the increased complication rate,^6^ leaving only autologous possibilities.

There is a wide variety of totally autologous reconstructive methods including lower abdominal flaps, gluteal perforator flaps (superior and inferior), thigh flaps (transverse gracilis, profunda artery perforator, anterolateral, lateral transverse), peri-iliac (Reuben's flap) and LD. However, the usefulness of these techniques is limited in thin and/or nulliparous patients because the lack of excess tissue means that any resulting reconstruction will be of inadequate volume^7^ or necessitate the use of complex microvascular surgical techniques^8^ or simultaneous ipsilateral fat grafting.^9^ The LD flap option in its extended or “total autologous” form provides an option by increasing available reconstructive volume by utilizing the additional back fat deposits of the scapular and parascapular areas, as well as lumbar fat.^10^^-^12

Initially, the totally autologous latissimus dorsi (TALD) flap was thought best for, and indeed found most utility in, women with higher BMIs because they had large fat deposits.^1^^,^^13^^,^^14^ There is little literature on the totally autologous reconstruction of small breasts, especially with the LD flap. It was therefore the objective of this study to review our use of the TALD flap in patients with breast cancer with lower BMIs and small breast volumes.

## PATIENTS and METHODS

Six mastectomy patients with lower than average BMI who underwent a TALD flap immediate reconstruction (ie, without implants) were identified from logbook records of a single surgeon (C.M.M.). For various reasons, patients did not wish to have reconstruction with implants. They were deemed by the surgeon to have sufficient fat deposits on their backs to match the volume of the contralateral breast. The patients’ medical and photographic records were accessed to identify patient age at reconstruction, BMI, breast cup size, surgical indication, mastectomy weight, adjuvant treatment, and outcome. The weight of the LD flap was not recorded because of lack of suspension scales and because of their attendant inaccuracy. The outcome was assessed both subjectively and objectively. An objective assessment of satisfaction with the cosmetic result was determined by whether any additional surgical interventions were performed or requested, whereas subjective assessment of breast symmetry was made by the authors using standardized independently produced medical photographs. The aesthetic outcomes were also objectively quantified using the Breast Conservation (BCCT.core) software, initially developed for assessing the results of breast conservation surgery. Frontal photographs of each patient were assessed preoperatively and postoperatively using the software that assesses the breasts across 3 main parameters: symmetry, color, and scar (if present).^15^^,^^16^

### Surgical technique

All patients were operated on in the lateral decubitus position simultaneously with the mastectomy. The ipsilateral back was infiltrated with standard liposuction fluid (Ringer's lactate; each liter containing 1 mg of epinephrine and 30 mL of 1% lidocaine) to reduce intraoperative blood loss and facilitate the dissection. After incision around the skin paddle, the skin flaps were raised just below Scarpa's fascia to maximize harvest of fat overlying the muscle. The dissection was extended beyond the boundaries of the LD muscle belly anterolaterally, inferiorly, and superiorly to increase the fat harvest similar to previous descriptions.^17^ Following inclusion of these extra fat deposits, the muscle flap was raised in a standard fashion preserving the thoracodorsal nerve and the “crossing branch” of the thoracodorsal vessels to the serratus anterior muscle. The former was preserved to minimize postoperative denervation muscle atrophy and thus reduction in resultant breast volume. The tendon of the LD muscle was divided in all cases to facilitate flap transposition and optimize its inset.

The flap was transposed anteriorly to the mastectomy pocket via a high tunnel while carefully avoiding pressure on or twisting the vascular pedicle. The flap was then shaped with 2/0 PDS sutures and inset to the pectoral fascia starting at the inframammary fold. This technique did not include fat grafting to increase the volume of the flap.^7^

Suction drains were used for both the donor and recipient sites (subflap and subcutaneously in the reconstructed breast).

### Case Reports

#### Patient 1

**Figure 1:**
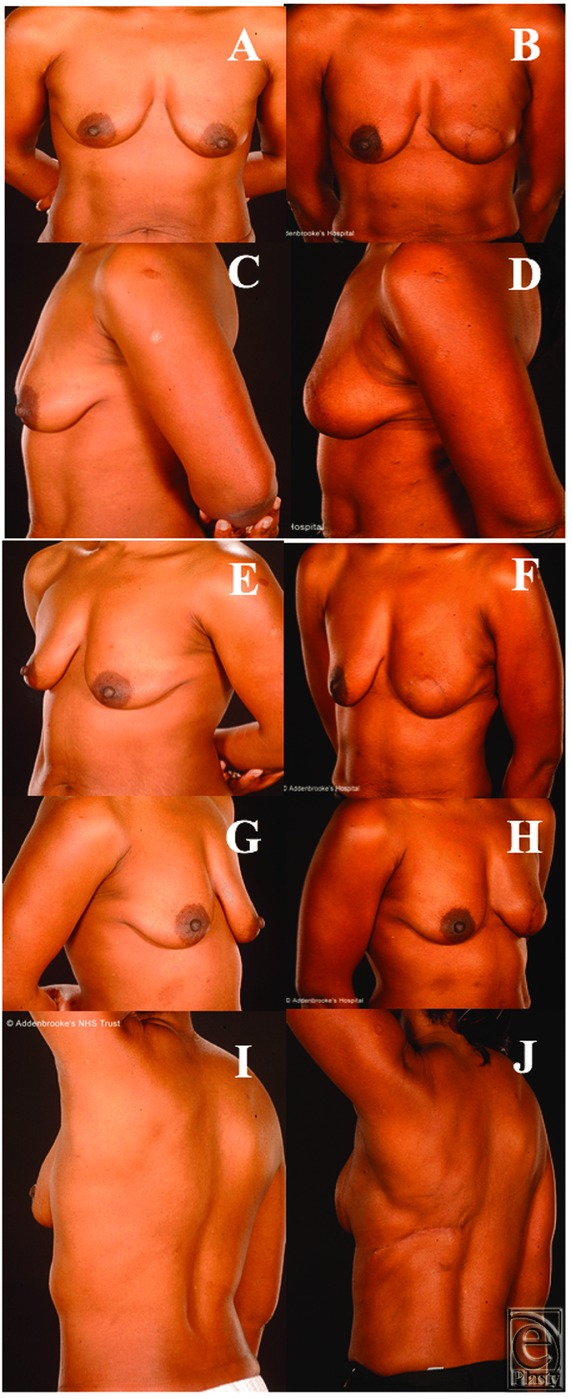
Patient 1, a 52-year-old woman with extensive ductal carcinoma in situ who under-went a left mastectomy and TALD reconstruction 1 year later. Note the excellent postoperative symmetry. Photographs prior to nipple reconstruction (a, c, e) and after TALD reconstruction (b, d, f). TALD indicates totally autologous latissimus dorsi.

A 52-year-old female patient with a BMI of 25 (1.5 m, 56 kg) and a 30A bra cup size was diagnosed with extensive intermediate- to high-grade ductal carcinoma in situ (DCIS). The breasts exhibited a degree of ptosis (Figs 1*a* and 1*c*). Her back tissue was evaluated as borderline adequate for an autologous LD flap. The mastectomy weight was 192 g. Her reconstruction was successful with excellent cosmetic results (Figs 1*b*, 1*d*, and 1*f*). She did not require postoperative adjuvant therapy.

#### Patient 2

**Figure 2:**
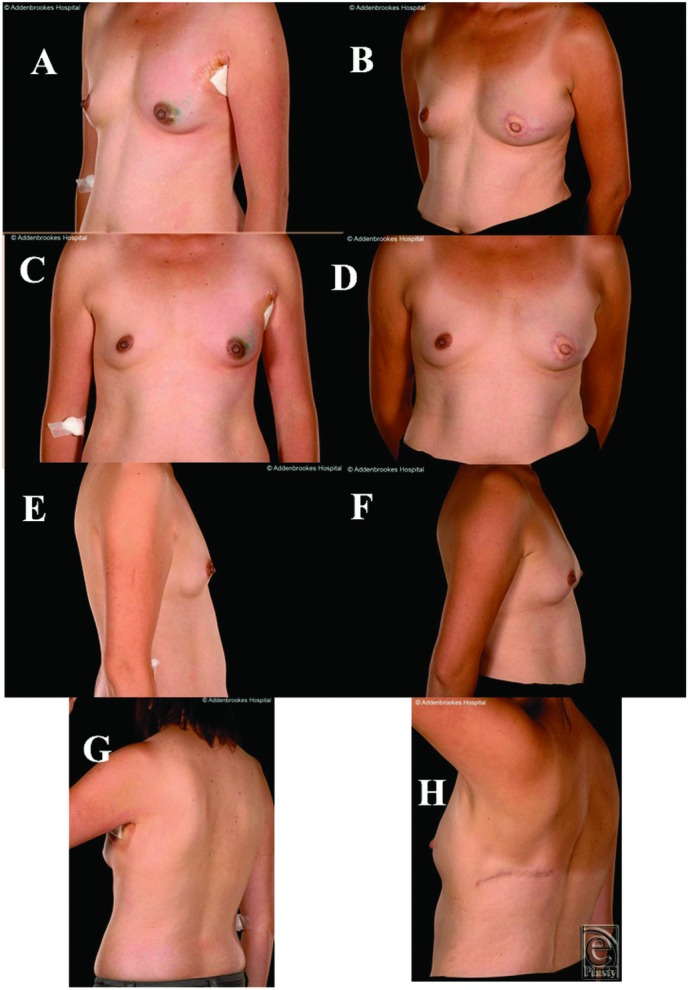
Patient 2, a 40-year old female with A cup size breasts and body mass index of 20, shown preoperatively (a, c, e) and 1 year following immediate left breast reconstruction with a totally autologous latissimus dorsi flap (b, d, f) and nipple-areolar reconstruction with acceptable cosmetic results.

A 40-year-old woman with multifocal DCIS (one focus grade II measuring 10 mm, and the other 30 mm and of a high grade) in her A cup-sized left breast (BMI = 20 kg/m^2^) opted for the TALD procedure. There was insufficient tissue on the buttocks and thighs, and abdominal skin closure would have been too tight for an abdominal flap. The cosmetic results (Figs 2*b*, 2*d*, and 2*f*) were assessed as acceptable, and she suffered no complications from the mastectomy (91 g of breast tissue removed) or the simultaneous sentinel lymph node biopsy.

#### Patient 3

**Figure 3:**
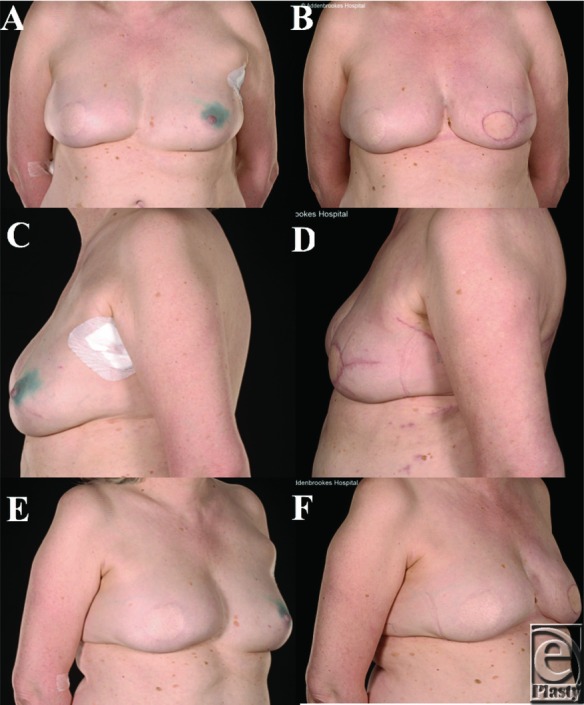
Patient 3, a 54-year-old woman with a body mass index of 26 and 36A/B cup-sized breasts, undertook a left breast latissimus dorsi reconstruction as demonstrated in the preoperative (a, c, e) and postoperative (b, d, f) photographs. She previously had a right skin-sparing mastectomy and reconstruction with a pediceled TRAM flap.

A 54-year-old woman (BMI 26; bra cup size of 36A/B) presented with a multifocal invasive ductal carcinoma (15-mm grade III focus and another at 6-mm grade II) with associated DCIS; bringing the total tumor size to 85 mm. Ten years earlier, she had undergone a skin-sparing mastectomy and reconstruction with pediceled TRAM flap for a contralateral cancer (Figs 3*a*, 3*c*, and 3*e*). Therefore, the abdominal tissue was no longer available for the reconstruction and there was inadequate tissue on the inner thigh, upper buttock, and lower buttock. Furthermore, considering the size and shape of her TRAM-reconstructed breast, it was deemed that reconstruction with an implant would not produce a good enough cosmetic result to match it. She underwent TALD flap reconstruction (which during surgery was successfully converted to a free flap after accidental avulsion of the vascular pedicel during flap transposition). The mastectomy weight was 601 g. She was very pleased with the cosmetic outcome, which was assessed as acceptable (Figs 3*b*, 3*d*, and 3*f*).

#### Patient 4

**Figure 4:**
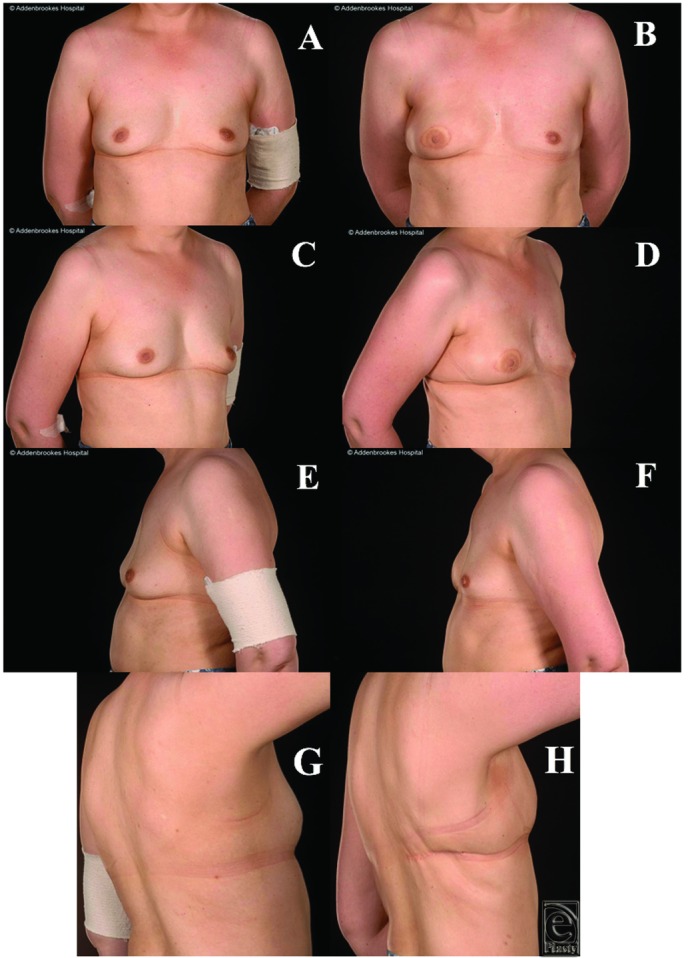
Patient 4, a 45-year-old woman with 36B cup size breasts and body mass index 27, shown preoperatively (a, c, e,) and 1-year after reconstruction (b, d, f). The nipple reconstruction has produced good cosmetic results, and there is reasonable donor site scarring. The breast mounds on the left-lateral view (e, f) highlight that there is more than adequate volume to match the opposite breast.

A 45-year-old nulliparous woman with a BMI of 27 and bra cup size of B was scheduled to have a mastectomy and axillary clearance surgery of the apical nodes after being diagnosed with a 40-mm grade III invasive ductal carcinoma. Considering her small breast size, the DIEP flap was considered too large a procedure while her planned adjuvant RT made implants a less favorable option. Adequate skin and fat were present on the back and inner thighs. When presented with the options of a TALD or TUG (transverse upper gracilis) flap reconstruction, the patient opted to have the former. Her mastectomy weight was 140 g. The cosmetic results were excellent (Figs 4*b*, 4*d*, and 4*f*), and there were no postoperative complications.

#### Patient 5

At 66 years of age, the oldest of the patients in this series and with the lowest BMI (20; 1.76 m, 63 kg) and a 30D bra cup size was diagnosed with a 26-mm grade III invasive carcinoma of no special type (NST, ER+ve, Her2-ve). She had undergone 2 prior excisional biopsies with incomplete margins and negative sentinel lymph node biopsy. The mastectomy weight was 220 g. Her cosmetic result was judged acceptable by the operating surgeon, and there were no complications.

#### Patient 6

A 35-year-old nulliparous woman with a BMI of 23 and bra cup size of 34C had been diagnosed with a multifocal grade III NST left breast cancer (with tumors measuring 23, 12, and 6 mm). She also requested a risk-reducing mastectomy. Several viable bilateral reconstructive options were fully discussed with the patient during her neoadjuvant chemotherapy, including an expandable implant combined with ADM support (despite future planned RT), extended DIEP flap (DIEP + DCIA fat), and bilateral LD flaps. The mastectomy weights were 244 and 206 g (left and right, respectively). She developed minor (superficial) donor site wound dehiscence, which was successfully treated with dressings and direct closure under local anesthetic. The postoperative cosmetic results were excellent.

## RESULTS

The patient clinicopathological characteristics are shown in Tables 1 and 2. Of the 6 patients (median age = 49 years; range, 35-66 years), all had normal but lower than average BMIs, and 5 possessed small breasts as confirmed by cup size or mastectomy weight (<350 g).^2^ Despite a BMI of 26 and a mastectomy weight of 650 g, one patient in whom a TALD flap was the only totally autologous option was also included. The mastectomies were performed for operable breast cancers comprising invasive carcinoma (n = 3) and extensive high-grade DCIS (n = 3). Patient 6 also had a contralateral risk-reducing mastectomy. Four had axillary surgery (2 sentinel lymph node biopsies; 2 axillary clearances), whereas 3 went on to receive adjuvant postoperative RT. The reason for a totally autologous reconstruction was due to patient choice, as well as likely requirement for postoperative RT in 3 patients (Table 3). There were no major postoperative complications, such as major wound dehiscence, as noted by other authors^10^ and no donor site aesthetic concerns.

**Figure 5:**
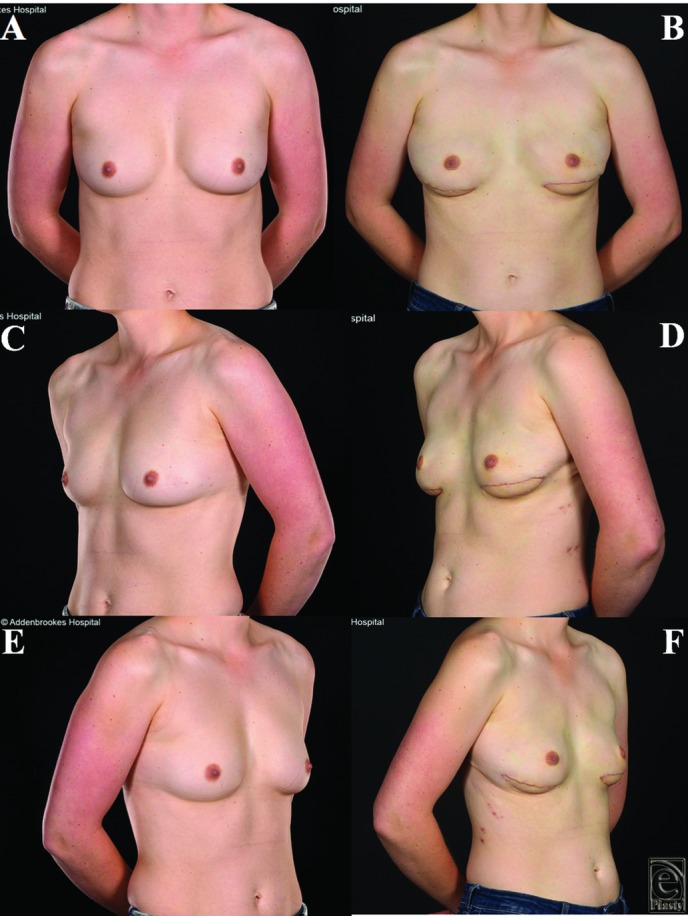
Patient 6, this 35-year-old woman with 34C cup size breasts and a left multifocal high-grade carcinoma of no specific type underwent bilateral nipple-sparing mastectomies (left therapeutic, right risk-reducing) and immediate reconstruction with bilateral latissimus dorsi flaps. The preoperative (a, c, e) and postoperative (b, d, f) appearances demonstrate that excellent cosmetic results were obtained.

All were satisfied with their reconstructive outcomes. For 5 of the patients, after minimum oncology follow-up of 5 years, none developed disease recurrence (the other is still within this 5-year window). Objectively, none of the 6 patients who underwent this procedure had any form of revision of the reconstructed breast such as lipofilling/prosthetic augmentation or contralateral balancing surgery to the other breast such as mastopexy, or breast reduction or liposuction. Subjectively, the reconstructions provided acceptable or excellent cosmetic results (Figs 1-5). The subjective assessment of symmetry by the reconstructive surgeons also demonstrated no significant difference between sides postoperatively (Figs 1-5). Quantitative assessments of symmetry on BCCT.core software demonstrated that the assessed symmetry of the breasts had been maintained or improved by the procedure in all evaluated patients (Table 4).

## DISCUSSION AND CONCLUSION

The use of the TALD flap in obese patients is well established.^11^^,^^13^^,^^18^ This is because such patients have sufficient fat deposits on their backs to match reconstruction of the large breasts often found in this group of patients. However, it is rarely thought of, let alone used, in thin patients. This is also the case in the Cambridge Breast Unit, in which, even before the introduction of ADMs in 2013 with consequent reduction in overall LD flap reconstructions, this technique was used in less than 5% of all LD flap total breast reconstructions. The present review of a small, single-operator series, however, found that even in thin patients with little excess back fat tissue, this totally autologous treatment option was not only viable but also gave very good and well-accepted cosmetic results.

Most thin patients have small breasts and are usually offered implant-based reconstruction either alone or in combination with an LD flap. The reason for this is the lack of fat deposits to match the patient's breast even when it is small. Our experience with postmastectomy TALD reconstruction in small-breasted patients with relatively low BMIs and low amounts of subcutaneous and other fat deposits is a positive one, with no patient experiencing any major postoperative complications and no patient undergoing any additional symmetrizing contralateral procedure or ipsilateral adjustments such as lipofilling or implant augmentation. These objective and definitive endpoints indicated patient and surgeon satisfaction with the cosmetic outcome.

The TALD flap was successfully employed for a variety of indications including DCIS and invasive cancer and in patients who later went on to receive postoperative adjuvant irradiation of their reconstructed breasts. None of these underwent further adjustment of their reconstructed breast such as lipofilling. The main shortcoming of the TALD flap in non-obese patients is that the volume of the tissue transferred may be insufficient and therefore require lipofilling to augment the reconstructed breast volume or shape either at the time of immediate reconstruction^7^^,^^9^ or subsequently.^19^^,^^20^ There is also the additional problem of muscle atrophy that besets all LD flaps whether they are used with or without an implant. In our study after an average follow up of more than 5 years, none of the patients have required or requested adjustment or balancing surgery, further confirming that the reconstructions were adequate and the fat transferred provided sufficient tissue. From a technical standpoint, it is important to make the reconstructed breast slightly bigger than the opposite breast, as there is a small and inevitable muscle atrophy despite preserving the thoracodorsal nerve.

While totally autologous reconstruction in the thin patient could be considered a contradiction, the outcomes for our patients were cosmetically acceptable and thus the TALD, in this small patient group in the hands of a low-volume operator, was a safe and viable treatment option that should be given more consideration, although it is not the typical indication for this form of reconstruction. In 2 of the patients, symmetry (as measured by the software) had improved postoperatively. We were able to reconstruct this challenging group of patients (made up of thin, small-breasted women) with the TALD flap without recourse to lipofilling or complex microvascular surgery such as bipedicled free flaps.^8^ This enabled us to avoid the use of implants that most patients had objected to on the grounds of “phobia” of foreign materials in the presence of cancer and fear of the effect of RT on an implant-based reconstruction despite our unit's acceptable capsular contracture rates in patients with immediate LD flap reconstruction following adjuvant radiotherapy.^21^ It has been reported by the Canniesburn group that the fat of the LD (as in the TALD flaps) withstands postoperative irradiation very well.^22^ The TALD flap is therefore particularly useful in patients in whom adjuvant RT is planned and decline implants or major reconstructive surgery. This has also been the experience of others.^23^

Furthermore, the TALD procedure can be considered a less complex operation, as it does not involve complex microvascular surgery—as required in a bipedicled DIEP procedures^8^ or TUG flaps. For patients who may want to minimize hospital stay, such as those with dependents or employment commitments, and so would like a simpler procedure, this makes the TALD an attractive option.

More recently, ADMs have been proposed as important adjuncts to implant-only immediate reconstruction when RT is planned, as ADMs reportedly reduce the risk of RT-induced severe capsular contracture.^24^ This is a useful way to enable thin patients with breast cancer to have implant-based reconstruction despite the planned RT. The biggest drawback of this approach is the high cost of the ADMs. In contrast, the TALD flap offers distinct cost advantages, as it involves no implant or ADM use. This is very useful in low-resource environments^23^ or in health care systems under financial constraints such as the British NHS. In addition, even if there was some reduction in volume postoperatively resulting in significant asymmetry, the TALD can be reliably augmented by fat grafting,^7^^,^^25^ thus still avoiding the use of implants altogether even in unfavorable indications. Lipofilling thus widens the utility and indications of the TALD to include patients with borderline amounts of fat.

In conclusion, the TALD flap is a viable alternative for autologous tissue-only breast reconstruction after mastectomy for cancer in selected thin patients with a relative balance between their breast size and the amount of fat deposits on their backs. Although applicable to only a small and select group of patients, this technique provides a valuable option when implant reconstruction is to be eschewed. It is useful to consider this often-forgotten and often-neglected option when faced with this challenging group of patients. However, we feel that it is perhaps an underused, or under-thought of, option in the reconstructive armamentarium of the breast plastic surgeon. With the advent of lipofilling as a reliable technique to enhance the breast volume, this flap should be considered more often than it is currently.

## Figures and Tables

**Table 1:** Summary of patient clinical data*

			Cup	Breast	Adjuvant		Subjective	
Patient	Age	BMI	size"	weight^†^	therapy	Symmetrization	assessment	Complications
1	52	25	30A	192 g	None	None	Excellent	None
2	40	20	A	91 g	Tamoxifen	None	Acceptable	None
3	54	26	A/B	601 g	Chemo + RT	None	Acceptable	Pedicle transection^‡^
4	45	27	36B	140 g	Chemo + RT	None	Excellent	None
5	66	20	30D	220 g	None	None	Acceptable	None
6	35	23	34C	225 g**	Chemo + RT	None	Excellent	Superficial WD

*BMI, indicates body mass index; RT, radiotherapy; Chemo, chemotherapy; and WD, would dehiscence on the noncancer side.

"“Cup Size” is as reported by patients on direct questioning.

^†^Mastectomy weight in the theater of cancerous breast (may include axillary clearance).

**Mean of left and right breast.

^‡^Complete intraoperative avulsion of the vascular pedicle—successfully converted to a free flap (free latissimus dorsi flap).

**Table 2:** Oncological details*

			ER	Her2 R	Axillary
Patient	Histology	Tumor size	status	status	surgery
1	Extensive intermediate- to high-grade DCIS	42 mm	N/A	N/A	None
2	Grade II invasive cancer + high-grade DCIS	10-mm IDC + 30-mm DCIS	+	−	SLNB
3	Grade III IDC + grade II IDC + DCIS (total tumor = 85 mm)	15-mm grade III + 6-mm grade II + 64-mm DCIS	+	−	ANC
4	Grade III IDC	40 mm	+	−	ANC
5	Grade III invasive cancer NST	26 mm	+	−	None
6	Multifocal grade III invasive cancer NST	23 mm + 12 mm + 6 mm	+	−	SLNB

*DCIS indicates ductal carcinoma in situ; N/A, not available; IDC, invasive ductal carcinoma; NST, no special type; and SLNB, sentinel lymph node biopsy; ANC, axillary node clearance.

**Table 3:** Clinical indications for LD flap reconstruction

Indications for totally autologous LD flap*	n (%)
Small breast	6 (100)
Refusal to accept implants	6 (100)
Planned radiotherapy	4 (66)
Inadequate or unavailable abdomen	3 (50)
Nulliparity	3 (50)
Refusal to have a large or complex operation	1 (16.5)
Old age (>65 y)	1 (16.5)

*LD indicates latissimus dorsi.

^†^Some patients had more than 1 indication.

**Table 4:** Subjective (clinical) and objective (BCCT.core) assessments of breasts

	Postoperative	Preoperative	Postoperative
Patient	subjective	objective	objective
1	Excellent	Good	Good
2	Acceptable	Good	Excellent
3	Acceptable	Good	Excellent
4	Excellent	Good	Good
5	Acceptable	…*	…
6	Excellent	Excellent	Excellent

*The patient did not attend for postoperative photographs.
